# Adaptation of influenza viruses to human airway receptors

**DOI:** 10.1074/jbc.REV120.013309

**Published:** 2020-11-22

**Authors:** Andrew J. Thompson, James C. Paulson

**Affiliations:** 1 Department of Molecular Medicine, Scripps Research, La Jolla, California, USA; 2 Department of Immunology & Microbiology, Scripps Research, La Jolla, California, USA

**Keywords:** influenza virus, cell surface receptor, sialic acid, neuraminidase, glycoprotein, hemagglutinin, pandemic, HA, hemagglutinin, HAE, human airway epithelial, IAVs, influenza A viruses, NA, neuraminidase, RBS, receptor-binding site

## Abstract

Through annual epidemics and global pandemics, influenza A viruses (IAVs) remain a significant threat to human health as the leading cause of severe respiratory disease. Within the last century, four global pandemics have resulted from the introduction of novel IAVs into humans, with components of each originating from avian viruses. IAVs infect many avian species wherein they maintain a diverse natural reservoir, posing a risk to humans through the occasional emergence of novel strains with enhanced zoonotic potential. One natural barrier for transmission of avian IAVs into humans is the specificity of the receptor-binding protein, hemagglutinin (HA), which recognizes sialic-acid-containing glycans on host cells. HAs from human IAVs exhibit “human-type” receptor specificity, binding exclusively to glycans on cells lining the human airway where terminal sialic acids are attached in the α2-6 configuration (NeuAcα2-6Gal). In contrast, HAs from avian viruses exhibit specificity for “avian-type” α2-3-linked (NeuAcα2-3Gal) receptors and thus require adaptive mutations to bind human-type receptors. Since all human IAV pandemics can be traced to avian origins, there remains ever-present concern over emerging IAVs with human-adaptive potential that might lead to the next pandemic. This concern has been brought into focus through emergence of SARS-CoV-2, aligning both scientific and public attention to the threat of novel respiratory viruses from animal sources. In this review, we summarize receptor-binding adaptations underlying the emergence of all prior IAV pandemics in humans, maintenance and evolution of human-type receptor specificity in subsequent seasonal IAVs, and potential for future human-type receptor adaptation in novel avian HAs.

Over the last 100 years, four human influenza A virus (IAV) pandemics resulted from the introduction of avian or avian/swine influenza virus strains that could transmit between humans and rapidly disseminate in an immunologically naïve population (1). In each case, as the novel pandemic virus adapted to its new host, and as host immunity became more widespread through natural infection and vaccination, it became a seasonal strain that evolves adaptive mutations in response to immune selective pressures over time (1, 2). The primary immunogens of influenza virus are the two surface glycoproteins, hemagglutinin (HA) and neuraminidase (NA), which also play key roles, respectively, in the binding to and release from receptors on airway epithelial cells, which are sialic acid (Neu5Ac) containing glycans (sialoglycans) on cell surface glycoproteins and glycolipids (3, 4) (Fig. 1). Viral HA initiates the infection cycle through specific binding to host-cell sialoglycans while NA acts at its conclusion, allowing newly synthesized virus particles (still bound to the cell surface by HA) to be released through hydrolysis of sialic acid linkages. Viral HAs generally exhibit specificity for sialic acid linkages predominately found in the species they infect, and for cross-species infection must adapt to the sialoglycans of the new host. As a consequence of avian influenza virus adaptation to human transmission, there is a characteristic change in the binding specificity of the virus HA from α2-3-linked “avian-type” receptors (Neu5Acα2-3Gal) to α2-6-linked “human-type” receptors (Neu5Acα2-6Gal) (3, 4) (Fig. 1*B*). This change is widely considered to be the result of selection of mutations that favor recognition of α2-6-linked human-type receptors that are predominately expressed on human upper airway epithelial cells (5). Furthermore, the α2-6 receptor-binding phenotype has also been shown to be critical for efficient aerosol transmission of human viruses in ferrets, currently considered the optimal animal model of human influenza virus transmission due to similar expression of α2-6-linked (human-type) receptors on airway epithelial cells (6, 7, 8). These findings suggest that acquisition of human-type receptor specificity may be required for successful person-to-person transmission of influenza within the human population, and thus acquisition of human-type receptor specificity is now considered a risk factor in the evaluation of avian viruses for pandemic potential in humans (1, 9).

**Figure 1:**
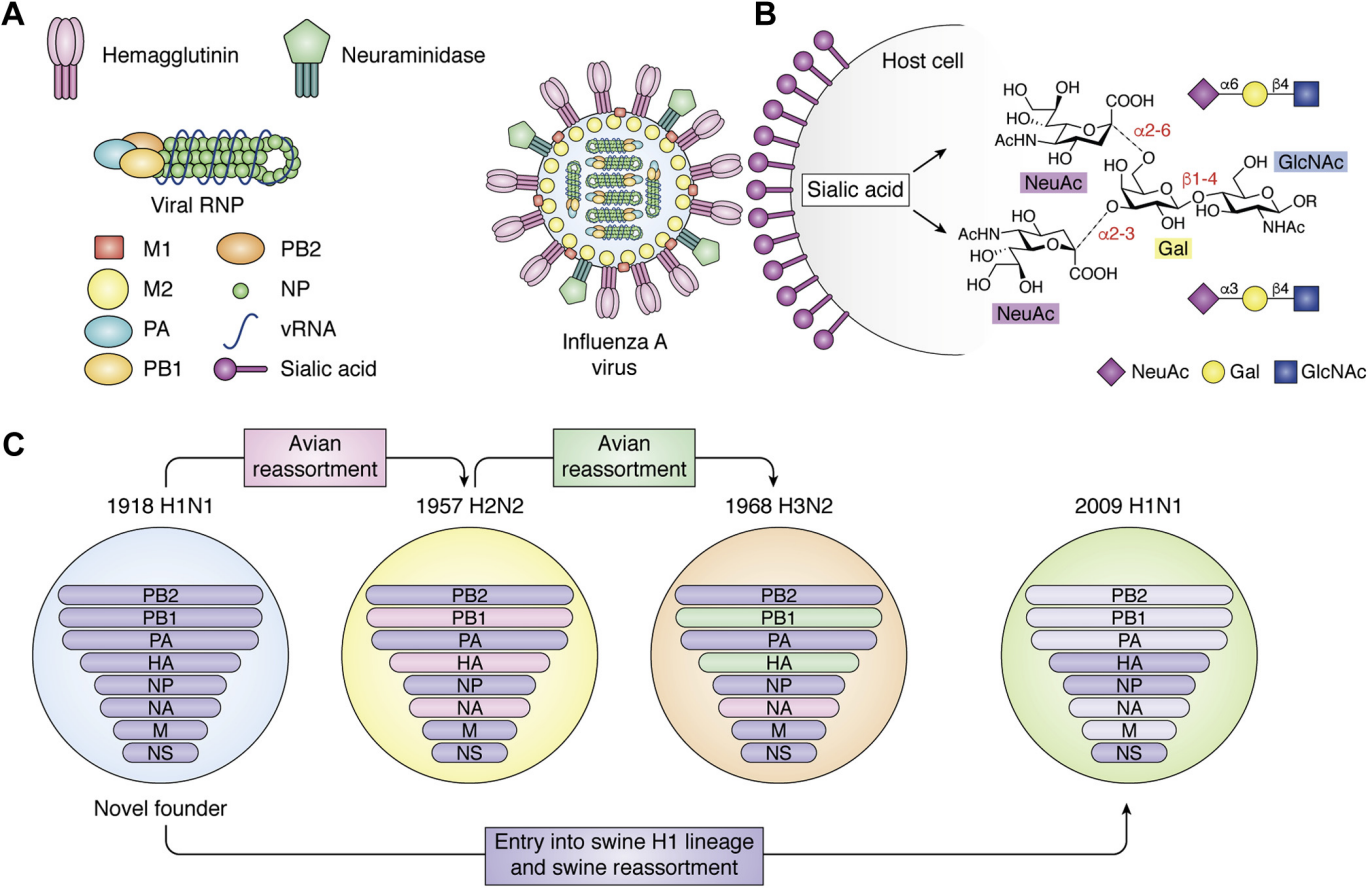
**Cartoon schematic of influenza A virus (IAV) virion structure, receptor binding, and gene reassortment.***A*, major viral proteins are shown labeled, including viral surface hemagglutinin, which mediates attachment to host cell sialic acid ligands. *B*, human and avian receptors differ by their receptor structures/chemistries featuring predominantly α2-6 and α2-3 linkages, respectively, in the upper airway. Structures shown illustrate that sialic acid can be linked to α2-3 or α2-6 to Galβ1-4GlcNAc representing common terminal fragments of glycans that occur in nature. *C*, schematic of IAV reassortments leading to novel pandemic viruses during the 20th and 21st centuries. 1918 H1N1 replaced all previously circulating human IAV strains and contributed significantly to all subsequent pandemics through reassortment with avian viruses. 1918 H1N1 also entered swine species, forming a distinct swine H1 lineage that eventually contributed genes to the novel 2009 H1N1 pandemic virus.

Here we review the molecular changes underlying adaptation of HA in human pandemic viruses to α2-6 (human-type) receptor specificity from α2-3 (avian-type) specificity in avian progenitors. We also highlight how human IAV HAs have retained human-type receptor specificity as the virus evolves during immune selection in the human population over time. With respect to avian virus strains that sporadically infect humans but have not yet acquired the ability to transmit among individuals (*e.g.*, H5N1, H7N9), we review what is known about the receptor specificity of these viruses and the potential for them to further adapt to human-type receptor specificity. Finally, it is now accepted that that IAVs require a balance between the counteracting receptor-binding and -destroying functions of HA and NA for optimal infection and to avoid “decoy” adsorption to extracellular mucins or glycoproteins (10, 11, 12, 13, 14). Therefore, since alterations in HA specificity must be compatible with the activity of viral NAs, we also briefly focus on accompanying NA activity variants, attempting to summarize current knowledge herein and referring the reader to several other excellent publications on this subject (11, 15, 16).

## Receptor specificity switch and drift of human pandemic-origin influenza viruses

IAVs have a negative-sense RNA genome subdivided into eight separate gene segments, encoding for between 10 and 14 individual protein components (1). Genes coding for the two major surface glycoproteins, HA and NA, are each found on separate gene segments (numbers 4 and 6, respectively). Among circulating avian and mammalian influenza viruses, 16 immunologically distinct HAs (H1–H16) and nine immunologically distinct NAs (N1–N9) have been identified, each of which can in principle be independently assorted into a large number of immunologically distinct influenza viruses (theoretically 144 different possible combinations) (1). Of these, just three combinations: H1N1, H2N2, and H3N2, account for the virus strains responsible for pandemic outbreaks in humans, with H1N1 occurring in 1918 and 2009, H2N2 in 1957, and H3N2 in 1968 (8, 17, 18, 19, 20, 21, 22). Emergence of the 1918 pandemic H1N1 strain has proved significant, not only in terms of disease severity, but also through its long-lasting influences on human IAV phylogeny. 1918 H1N1 replaced all previous IAV strains circulating in humans and established a new lineage of viral descendants where all subsequent human pandemic viruses have retained between three and five of its gene segments, acquired through reassortment as illustrated in Figure 1*C* (2, 23). To date, all newly emerged human pandemic IAVs have acquired a novel HA gene from an avian virus progenitor, with the exception of 2009 H1N1 where the novel H1 HA was derived from a swine IAV lineage originating from 1918 H1N1 (24) (Fig. 1*C*), suggesting that the role of HA as both a major antigenic and receptor specificity determinant comprises a key feature of host adaptation.

Comparison of the receptor-binding specificity of human pandemic viruses with corresponding HAs from avian viruses circulating at the same time has revealed in each case that pandemic strain HAs acquired specific adaptive mutations, increasing binding to human-type receptors, while simultaneously reducing or eliminating binding to avian-type receptors (8). Furthermore, evidence now shows a pattern of continued adaptation to human-type receptors in the years following introduction into the human population, including evolution of IAVs with increased receptor-binding avidity as well as linked alterations in NA activity, thus creating a defined balance in their counteracting receptor-engaging and receptor-destroying functions (10, 12, 13, 22, 25). This ability to adapt appears to have allowed human IAVs to simultaneously mutate to escape an immune response while maintaining their ability to bind receptors and transmit in the human population. In the following sections, we describe what is known about the adaptation of receptor specificity in human pandemic viruses.

### 1918 pandemic H1N1

**Figure 2:**
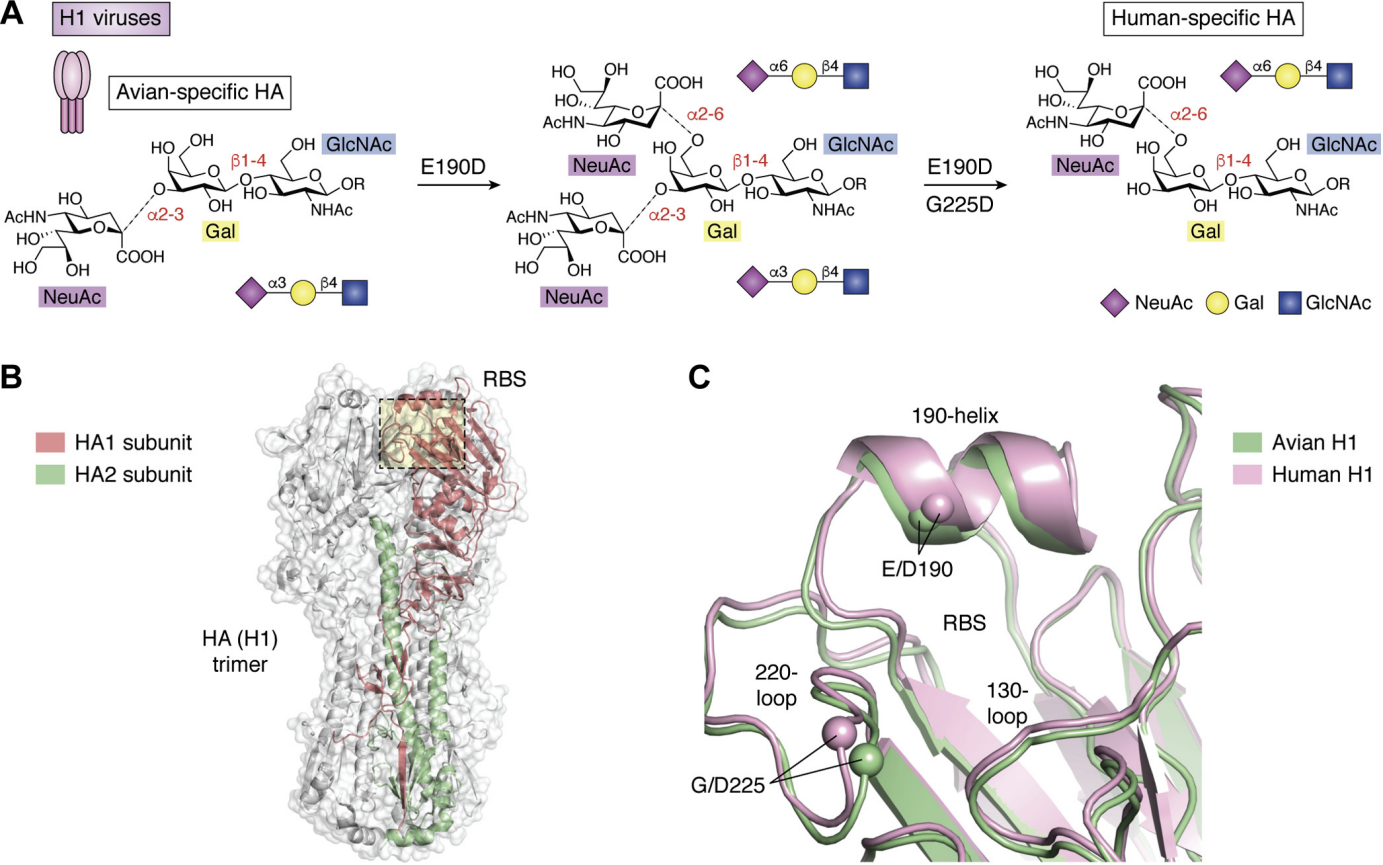
**Humanizing H1 amino acid variants and IAV hemagglutinin and receptor-binding site structures.***A*, effects of individual receptor-binding variants on viral receptor specificity that contributed to H1N1 entry into humans. *B*, overall structure of the HA (H1) hemagglutinin trimer shown as a transparent surface. HA1 and HA2 subunits from one protomer of the trimer are highlighted in *red* and *green*, respectively. *C*, architecture and key structural features of avian (*green*) and human (*pink*) H1. The locations of key amino acid positions are highlighted through depiction of Cα positions as enlarged spheres and *via* labeling of key residues and secondary structural features. Assembled in Pymol (Schrodinger LLC) using PDB IDs: 1RV0 and 1RVZ.

The 1918 H1N1 influenza pandemic remains the most devastating viral outbreak in modern history with severe respiratory-disease-associated fatality estimates for 1918 to 1919 extending into the tens of millions (estimated up to 50 million (2, 23) [https://www.cdc.gov/flu/pandemic-resources/1918-pandemic-h1n1.html], June 2020). Although the first live human influenza virus samples were not isolated until 1933 (6), the 1918 pandemic H1N1 strain has been successfully sequenced and reconstructed from preserved histologic specimens and autopsy samples from frozen human remains interred during the pandemic (26, 27). Comparison of these sequences with avian strains cocirculating at that time revealed that the human pandemic strains harbored two HA variants in the receptor-binding pocket, E190D and G225D (the standard H3 numbering convention is used here and throughout), that allowed for a switch in binding specificity from avian-type α2-3 to human-type α2-6 sialoside receptors (17, 28). Indeed, sequenced HA receptor-binding site (RBS) fragments from numerous independent samples revealed all pandemic viruses to contain E190D, which alone gave rise to mixed α2-3/α2-6 binding (17, 28, 29), while viruses containing both E190D and G225D showed strong α2-6 specificity (17, 28, 29) (Fig. 2*A*) with a potential preference for longer human-type receptors (29), consistent with later observations for human H3N2 viruses (see below). Structural analyses (28, 30) have revealed these variants to influence receptor specificity *via* two slightly different but related mechanisms whereby mutations within the 190-helix, located at the top of RBS, influence stability and remove side chains causing potential hindrance to incoming human-type receptors, while 220-loop variants, located toward the RBS base, subtly influence preferential accommodation of α2-3 *versus* α2-6 linkages (Fig. 2, *B*–*C*). With minor exceptions, the E190D–G225D variant pair underlying the 1918 pandemic receptor specificity switch appears to have been well maintained in all pandemic-descended H1N1 viruses until the eventual disappearance of these strains in 1957.

### Reintroduction of seasonal H1N1 in 1977

Pandemic-descended H1N1 viruses containing both E190D and G225D circulated continuously in humans until 1957 when they were replaced by a new H2N2 pandemic virus (described in more detail below). However, in 1977, an H1N1 “Russian flu” emerged during the seasonal winter outbreak. Since sequencing analyses revealed the 1977 virus to be near-identical to H1N1 strains that had previously circulated around 1950, it has been surmised to have been reintroduced into the population *via* accidental laboratory release (31, 32, 33). Owing to their antigenic similarity to the 1950 H1N1 strains, post-1977 H1N1 viruses were viewed then and subsequently as novel “seasonal” influenza strains and continued to cocirculate with H3N2 viruses that were dominant during this period. This feature in particular has proved interesting, since until 1977 circulating human influenza viruses consisted only of a single strain, and it was assumed that newly emerging viruses typically replaced prior strains due to direct competition for the same host. Reasons for this apparent shift in viral ecology remain unclear; however, notably, all post-1977 seasonal H1N1 strains maintained the E190D–G225D receptor-binding variants and appear specific for the same human α2-6 receptors as H3N2 counterparts.

### 2009 pandemic H1N1

**Figure 3:**
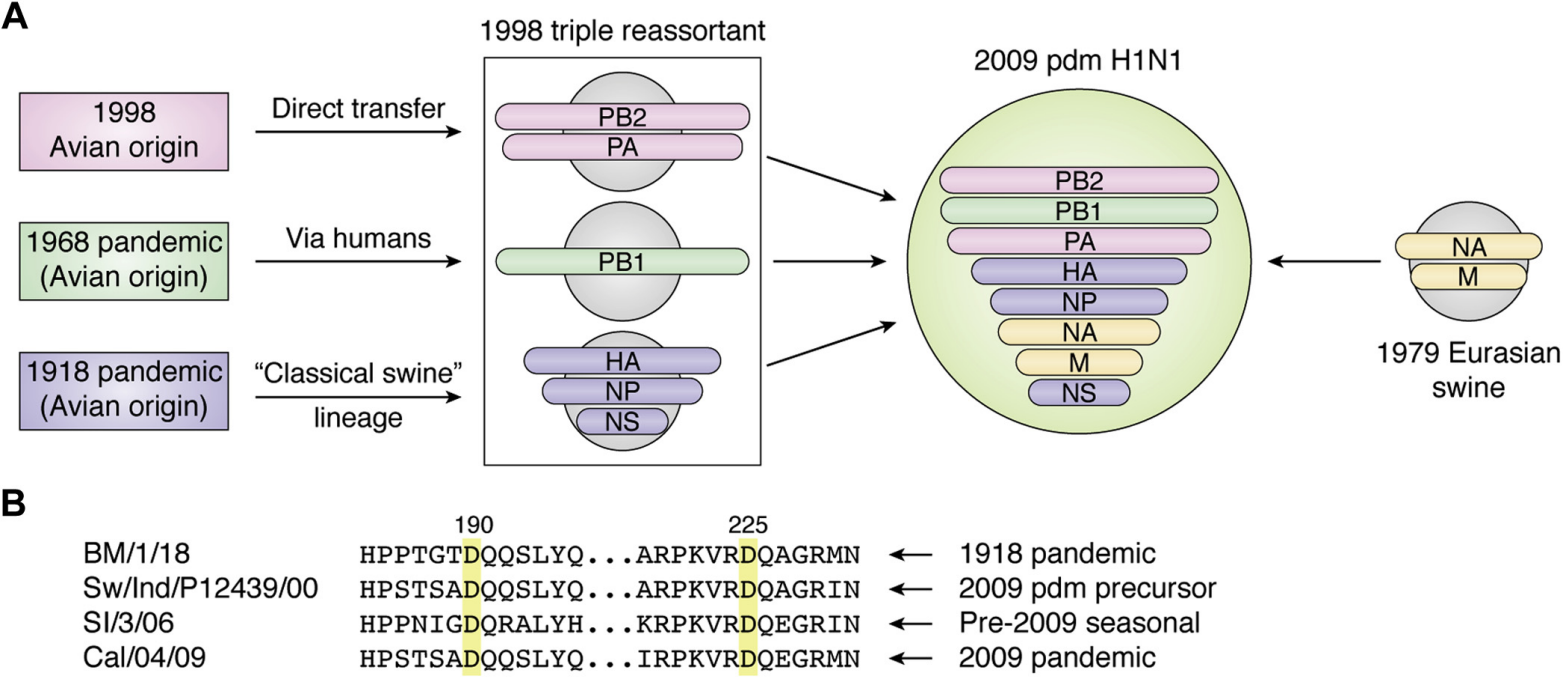
**Reassortment leading to emergence of the 2009 pandemic H1N1 virus.***A*, cartoon schematic of various swine lineages contributing gene segments to the novel virus (adapted from data in Garten *et al.* (2009) (24)). *B*, conservation of key humanizing receptor-binding variants in example viruses from the original 1918 pandemic, through the classical swine H1N1 lineage, and within both pre- and post-2009 human H1N1 strains. Strain abbreviations are: A/Brevig Mission/1/1918, A/swine/Indiana/P12439/2000 (nearest identified swine HA gene precursor to 2009 human pandemic strains), A/Solomon Islands/3/2006 (pre-2009 seasonal), and A/California/04/2009 (2009 pandemic).

In mid-2009, a novel H1N1 strain originating in Mexico and the southern United States (34, 35, 36, 37) began to spread widely in the human population and replaced all 1977-descended H1N1 viruses during the subsequent winter flu season. Since the 2009 outbreak was the first human pandemic to occur following technological revolutions in both molecular biology and nucleic acid sequencing, details of the viral genome sequence and likely phylogenetic origins were published quickly, revealing the novel strain to be a triple reassortant virus composed of genetic elements from three separate swine lineages (24, 37). 2009 H1N1 viruses were shown to be composed of viral polymerase genes (PB2, PB1, PA) from contemporary 1990s swine influenza, NA and M genes from older Eurasian swine lineages, and, interestingly, HA, NP, and NS genes from “classical” lineages tracing back to 1918 H1, but whose distinct evolution had previously confined them to pigs, with only sporadic zoonotic transfer into humans prior to 2009 (24, 37) (Fig. 3*A*). While the number of fatalities associated with 2009 H1N1 was reduced compared with previous pandemics (estimated at approximately 360,000 deaths worldwide), this outbreak shed new light on the potential dangers posed by viral reassortment since many of the 2009-strain genetic components had circulated for considerable time and appeared similar to prior human H1N1 strains (24, 37). In particular, the new pdm2009 H1 HAs had approximately 80% sequence identity at the amino acid level compared with prior seasonal H1 viruses, including the critical 190D and 225D substitutions required for human-type receptor specificity. Indeed, sequence alignments of post-1918 pandemic, pre-2009 seasonal, and swine precursor H1s reveal wide conservation of humanized receptor-binding variants suggesting that circulating classical swine-origin H1s had acquired specificity for human α2-6 receptors for quite some time prior to the pandemic outbreak (see Fig. 3*B*) (38). While acquisition of human receptor specificity is presumed to be a requirement for transfer into and transmission among the human population, alone it is not sufficient for full human adaptation. Rather, the 2009 pandemic virus appears to have acquired this property from the unique combination of several reassorted genes together (24). Indeed, it is well recognized that zoonotic avian viruses typically require adaptive changes within the viral polymerase complex in order to achieve successful replication within human cells. 2009 pandemic H1N1 viruses inherited the PB1 gene *via* a swine lineage that emerged in the late 1990s from human H3N2 strains (24, 37), a combination that may not have been prevalent or even possible at an earlier time, and which likely conferred significant host-adaptative potential.

Although early 2009 pandemic strains did appear well adapted in terms of human receptor specificity, several studies have shown that HA-binding avidities from the earliest isolates were very low, but rapidly evolved to higher avidity after initial circulation in humans (13, 39, 40). Several groups have also highlighted the importance of balanced HA/NA activities in human influenza viruses for this pandemic virus, and it appears likely that the H1 genetic lineage that gave rise to 2009 pandemic strains would not have been able to emerge without the combination of a complementary low-activity N1 gene, in this case emerging from a more distant Eurasian swine background (24, 37). Subsequent strains evolved from the pandemic now appear to have adapted as seasonal strains with improved HA receptor binding and NA activity (13, 39, 40).

### 1957 H2N2 pandemic

In 1957, a novel pandemic influenza strain thought to have originated in Japan circulated globally through the human population, rapidly replacing seasonal H1N1 strains that had persisted in humans since the 1918 pandemic. Subsequent genetic analysis revealed that human H2N2 strains arose through reassortment of established human H1N1 viruses with Eurasian lineage avian H2N2, leading to transfer of the PB1, HA, and NA genes (Fig. 1*C*) (41, 42). Thus, while the novel pandemic virus retained substantial similarity to 1918-descended strains internally, with five out of six internal genes conserved, the H2N2 viruses expressed completely novel HA and NA surface glycoproteins, leading to substantial disease and rapid spread among an immunologically naïve population.

The receptor specificity of early human H2N2 strains typically showed a mixture of binding to α2-6 human-type and α2-3 avian-type receptors, in contrast to avian H2 viruses that preferentially bound α2-3 avian-type receptors. Some of the earliest H2 isolates were found to have a mixture of two virus subtypes (*e.g.*, A/RI/5/57 and A/Japan/305/57) called the (+) and (−) subtypes (22). The (+) subtype was specific for α2-6 human-type receptors and strongly sensitive to inhibition of hemagglutination (HAI) by equine α2-macroglobulin in horse serum, a glycoprotein with α2-6-linked sialoglycans that are potent competitive inhibitors for human viruses (43). In contrast, the (−) subtype bound α2-3 avian-type receptors and was completely resistant to HAI by horse serum, the phenotype exhibited by avian H2 influenza viruses (22). Since receptor binding can drift during laboratory passage, particularly within chicken eggs, it is not known which subtype was dominant in the initial human infections, but appears likely that the pandemic spread would have been driven by the α2-6 human-type-binding (+) subtype, even if both (+) and (−) variants were able to cocirculate during this time. As H2N2 IAVs further adapted to human hosts, they acquired greater preference for α2-6 human-type receptors and remained sensitive to HAI by horse serum suggesting that this phenotype was strongly selected until the virus was eventually replaced by H3N2 in 1968. As H2 HAs adapted to increased human-type receptor specificity, the N2 NA was also shown to have acquired increased activity for cleavage of α2-6 human-type receptors (25), demonstrating coevolution of the two proteins to maintain an HA/NA balance as noted earlier for 2009 H1N1 strains.

**Figure 4:**
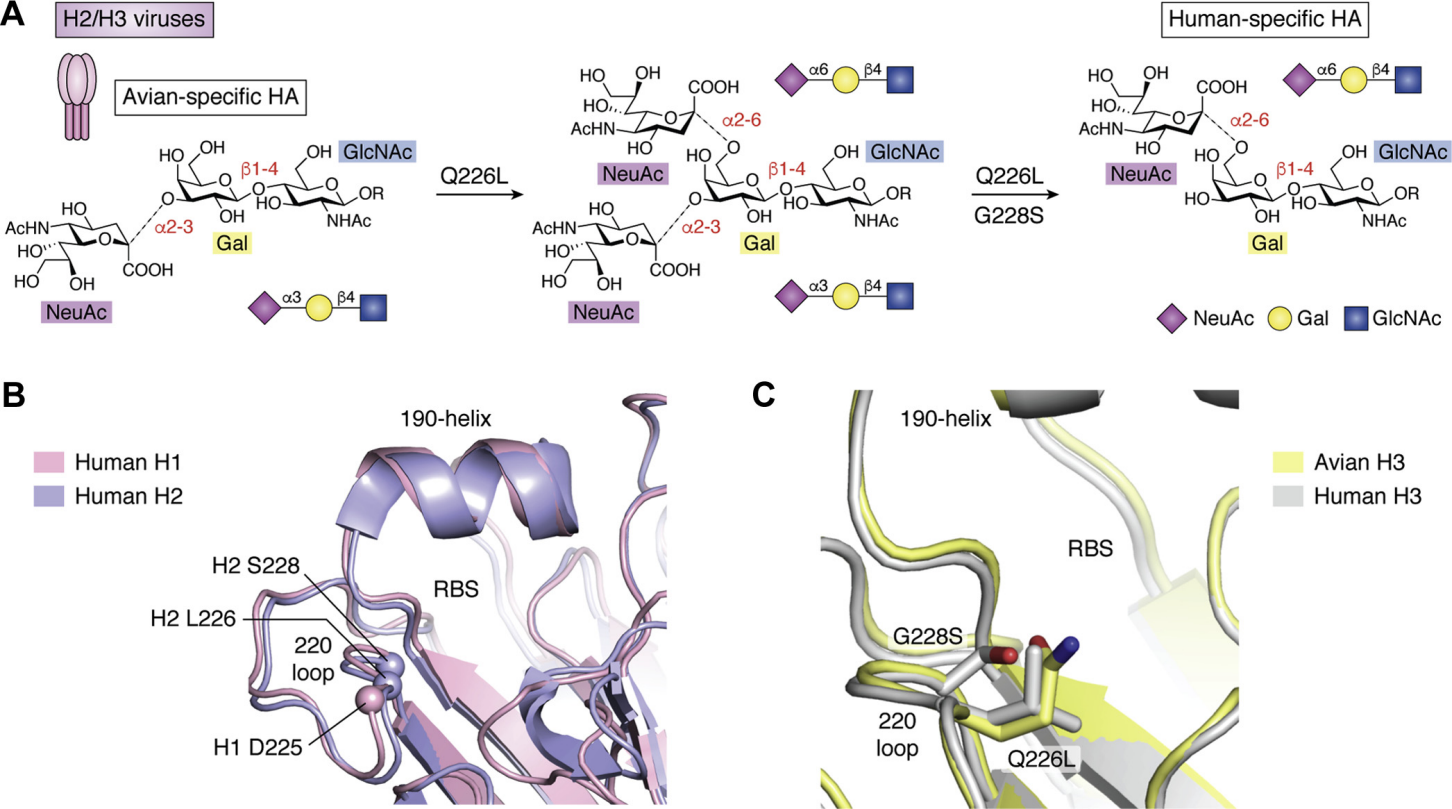
**Human-adaptive receptor-binding variants underlying the 1957 H2N2 and 1968 H3N2 pandemic viruses.***A*, similar to 1918 H1N1, H2 and H3 HAs feature a single dominant variant (Q226L) enabling binding to human-type receptors and a stabilizing covariant (G228S) that ensures specificity. *B*, architecture and key structural features of human H1 (*pink*) and human H2 (*lilac*). The locations of key amino acid positions are highlighted through depiction of Cα positions as enlarged spheres and *via* labeling of key residues and secondary structural features. The key 226 and 228 positions in H2/H3 lie in close proximity to D225 in human H1. *C*, enlarged overlay of the avian (*yellow*) and human (*gray*) H3 RBS. Panels (*B*) and (*C*) assembled in Pymol (Schrodinger LLC) using PDB IDs: 1RVZ, 3KU6, 1MQM, & 6TZB.

Sequence analyses of the HAs of cocirculating subtypes of 1957 H2N2 isolates and H2 avian viruses revealed that a pair of mutations in the (+) subtype, Q226L and G228S, corresponded to the acquisition of binding to human-type receptors and HAI sensitivity to horse serum (22) (Fig. 4). The importance of these amino acid substitutions individually and together was elegantly confirmed by solid-phase binding assays and analysis of X-ray crystal structures of the H2 HA in complex with sialic-acid-containing receptors (18, 19) (see final section for detailed structural analysis and discussion). It is notable that variants required for conversion of H1 HAs to human-type receptor specificity, E190D and G225D, are not found in human H2 isolates, demonstrating that while binding to α2-6 human-type receptors is selected for in human viruses, the solution for achieving this specificity can be different for different HA subtypes. Although quite distinct variants, the broad effect of Q226L/G228S appears similar to G225D in H1 (Fig. 4*B*), where adaptive variants act in close proximity to the Neu5Ac-Gal glycosidic linkage to promote binding to α2-6 receptors, while simultaneously decreasing preference for α2-3 through steric hindrance. Also similar to 1918 pandemic evolution is the apparently slow adaptation to human receptors in the immediate aftermath of the 1957 outbreak. Specifically, most human H2N2 viruses were found to possess Q226L, which confers mixed α2-3/α2-6 binding (comparable with the effect of E190D in H1; Figs. 2*A* and 4*A*), with prevalence of this variant spreading rapidly (18). However, evolution of the Q226L-G228S double variant, leading to stricter human-type specificity (comparable with G225D), was not widely observed until 1958 to 1959 onward (18). Retained partial binding to avian α2-3 receptors in H2N2 virus HAs may well reflect a requirement for NA balance, with data showing that NA activity did not adapt to cleave human receptors until considerable time after the pandemic (25).

### 1968 H3N2 pandemic

Approximately 10 years after the emergence of H2N2, in 1968, a third 20th-century pandemic influenza strain was introduced into and spread rapidly through the global human population. Originating in Hong Kong, the novel H3N2 strain evolved under highly similar circumstances to the H2N2 virus, *via* reassortment with contemporary avian strains, resulting in the transfer of a novel surface H3 gene and the polymerase gene PB1 (41, 42). Otherwise, the previously avian-acquired N2 gene and five remaining internal genes, PB2, PA, NP, M, and NS, were inherited from the H2N2 virus (previously acquired from H1N1) and remained unchanged, underscoring again the potential risk of comparatively minor reassortments that can lead to antigenically novel and highly virulent new strains. Interestingly, despite novel introduction from an avian source, the new pandemic H3 exhibited identical receptor-binding mutations, Q226L and G228S (Fig. 4*C*), to those in H2N2 strains and showed strong specificity for human-type receptors and sensitivity to HAI by horse serum from early in the pandemic outbreak (20, 21, 22, 44).

The importance of the Q226L mutation for binding α2-6 human-type receptors was initially demonstrated by growing a 1968 human H3N2 strain in eggs in the presence of horse serum, resulting in the selection of a receptor variant with a single L226Q mutation that bound avian-type receptors and was insensitive to HAI by horse serum (20, 45). Selection in the reverse direction was demonstrated by growth of the H3N8 avian virus precursor virus, A/duck/Ukraine/1/63 on human-type receptor modified MDCK cells resulting in selection of a human-type receptor variant with a single Q226L mutation that conferred human-type receptor specificity (45). However, while this single amino acid is sufficient for a receptor specificity switch of the avian virus HA to α2-6 human-type receptor specificity, it reverted back to avian-type specificity in a single passage in eggs (45). This observation and the coevolution of Q226L and G228S in both H2N2 and early H3N2 strains suggest that the G228S mutation, while playing a minor role in the switch in receptor specificity, provided stability for the Q226L mutation to persist.

### Receptor specificity drift in H3N2 seasonal strains

**Figure 5:**
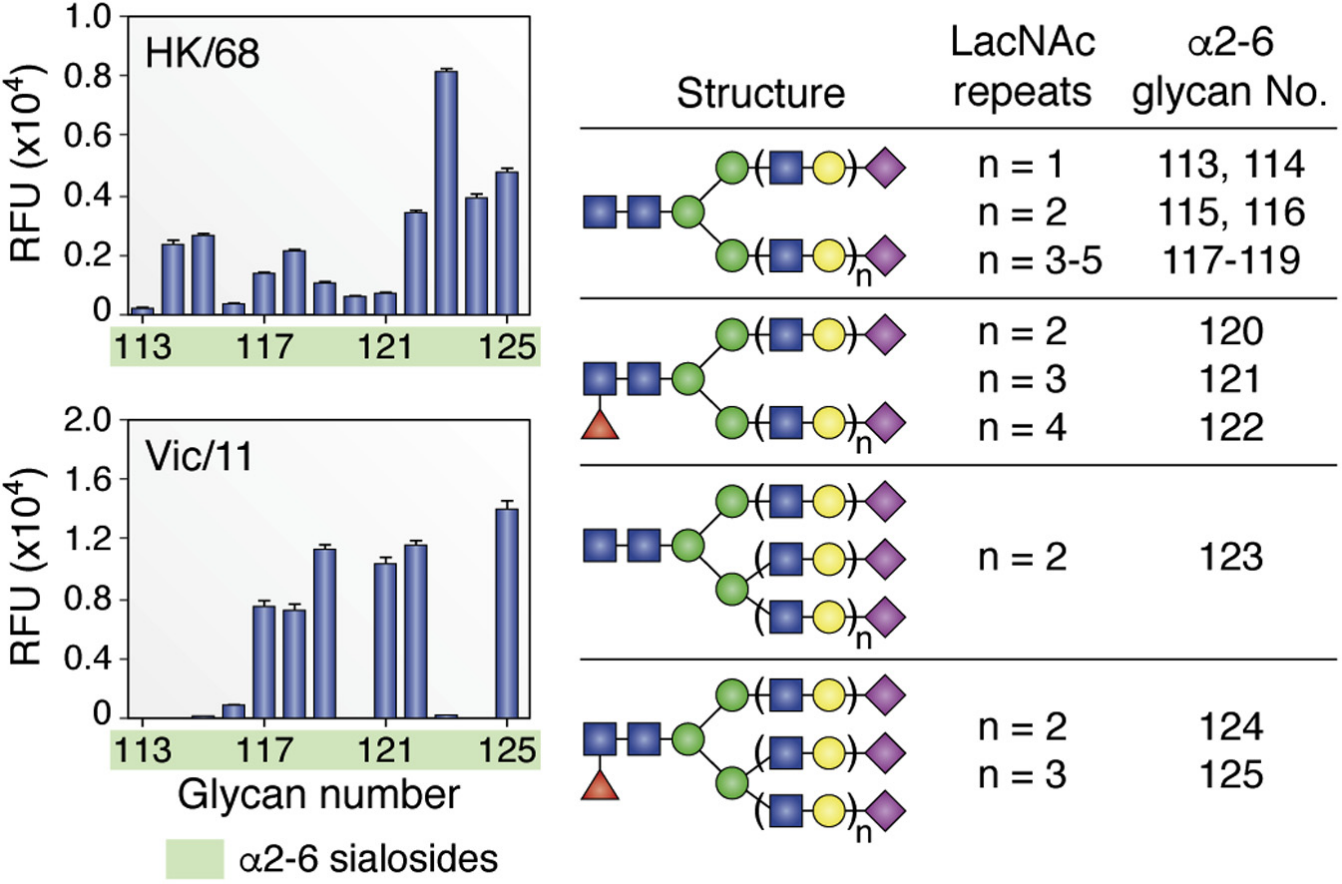
**Length-selective binding of contemporary human H3N2 viruses.** Glycan microarray segments are shown comparing binding of A/Hong Kong/1/68 (novel H3N2 pandemic strain) with A/Victoria/361/2011 (adapted human seasonal strain) to exclusively α2-6-linked N-glycans that vary only by length through the number of N-acetyllactosamine (LacNAc) repeats beneath the terminal sialic acid. Vic/11 binds exclusively to N-glycans featuring at least 3 LacNAc units. Note: for compounds 113 to 119, biantennary N-glycans, (LacNAc)_2_ and (LacNAc)_3_ structures are duplicated (114 & 115 and 116 & 117, respectively) with slightly different chemical attachment linkers. Figure adapted from data published in Peng *et al.* (2017) (49) with permission from Cell Press, see manuscript for further details.

Since the initial adaptation of H3N2 viruses to human-type receptors, they have circulated in the human population for over 50 years and over time have drifted to a more restricted receptor-binding specificity (46, 47, 48, 49, 50). This has become particularly evident during the last decade, where H3N2 isolates have proved increasingly difficult to recover in laboratory hosts and failed to hemagglutinate erythrocytes in standard hemagglutination titer assays. In several reports, H3N2 viruses isolated after 2003 were found to bind poorly to α2-6-linked sialoside receptors on glycan arrays and other solid-phase binding assays, leading to the conclusion that these viruses had lost specificity for human-type receptors (46, 47, 48). However, it subsequently became apparent that later H3N2 strains retained strong specificity for human-type receptors, but bound preferentially to a subset of extended sialic acid α2-6-linked glycans with additional N-acetyllactosamine (Galβ1-4GlcNAc) repeat sequences. HAs from these viruses appeared to lose binding to human-type receptors as a result of substantial but selective loss of avidity for “short” α2-6 glycans including the sialyl-N-acetyllactosamine trisaccharide (NeuAcα2-6Galβ1-4GlcNAc), commonly used in laboratory-based receptor assays (Fig. 5) (49).

This restricted specificity of the H3 HA for only a subset of human-type receptors has had significant impact on influenza research and vaccine development since it coincided with, and likely directly contributed to, the inability of more recent H3N2 strains to propagate efficiently in laboratory hosts such as MDCK cells and eggs. Furthermore, poor binding to erythrocytes used in hemagglutination and HAI assays to measure virus titers and determine inhibitory potency of antibodies in serum, respectively, has proved an equally significant limitation even when potential vaccine strains can be grown successfully. Mismatches in receptor specificity of IAVs for their natural receptors in humans and receptors present in laboratory hosts are not new (51, 52, 53, 54, 55), having been described in 1949 by Burnet upon passage of H1N1 viruses in eggs resulting in a switch from the “O” (original) to “D” (derived) specificity, as measured by acquisition of hemagglutination with chicken erythrocytes (51). For recent H3N2 viruses, specificity mismatches are particularly consequential since commercial vaccines are produced in eggs, and primary H3N2 isolates grow poorly in these hosts due to the absence of extended α2-6 sialic acid receptors found in humans (56, 57, 58). Indeed, it is well documented that N-linked glycans found on cells lining the amniotic membrane of chicken eggs contain both human type (α2-6) and avian type (α2-3) sialic acid linkages; however, these receptors are found exclusively on short trisaccharide extensions attached to the Man_3_GlcNAc_2_Asn core (59). As a result, contemporary H3N2 IAVs grown under these conditions experience a strong selective pressure in favor of receptor-binding adaptation, typically leading to selection of mutants with improved binding to shorter glycan receptors. A well-document example of this is highlighted in post-2014 H3N2 strains within clade 3C.2a (selected as Northern and Southern hemisphere vaccine strains in 2016–2017/2018 flu seasons). During 2014, clade 3C.2a strains circulating natively within humans gained an additional glycosylation site at position N158 within the HA head domain through widespread selection of the K160T variant, presumably due to adaptive antigenic drift. Presence of additional steric bulk above the H3 RBS appears to reinforce selectivity for extended human-type receptors, since H3N2 strains produced in eggs (though not within MDCK cells) for subsequent seasonal vaccines resulted exclusively in reversion to 160K, removing the N158 glycan and presumably enabling improved growth in eggs through binding to short glycan receptors (57). Analyses of antibodies produced in both humans and ferrets vaccinated with the egg-grown vaccine showed reactivity to mutant T160K HA, but poor reactivity and neutralization of viruses containing wild-type T160 HA (56, 57). CDC data shows that vaccine effectiveness (VE) against H3N2 strains has been steadily decreasing over the last several years (https://www.cdc.gov/flu/vaccines-work/past-seasons-estimates.html, June 2020), in part due to the highly evolved receptor specificity now prevalent within these strains. Thus, there is now significant concern that selection of receptor-binding mutants resulting from growth in eggs could further impact the efficacy of annual vaccines (56, 57, 58, 60).

An alternative and potentially more effective route to vaccine production in chicken eggs lies in large-scale cell culture, utilizing lines such as MDCK and its various derivatives. However, while passage of IAVs in MDCK cells does result in fewer HA mutations, effects of receptor mismatching on properties such as antigenicity can still be observed (61). This, together with the prohibitively increased cost of industrial-scale cell-culture-based manufacturing, has largely prevented the widespread adoption of cell-culture-produced seasonal IAV vaccines; however, significant interest remains in engineering improved cell lines with both lower mutation rates and enhanced viral yields to one day to tackle this problem. One early successful example of this approach from Matrosovich and colleagues engineered MDCK cells to increase NeuAcα2-6Gal glycan receptors by stably transfecting the cells with the sialyltransferase gene, ST6Gal1, responsible for synthesis of this linkage (62). The resulting cell line, MDCK-SIAT1, showed increased recovery of H3N2 viruses from patient swabs (62, 63). This approach has recently been extended by the Kawaoka group through similar overexpression of ST6Gal1 in MDCK cells, subsequent to removal *via* genetic knockouts of all ST3Gal sialyltransferase genes responsible for producing α2-3-linked avian-type receptors. The resulting cell line (hCK) maximizes the capping of glycans with NeuAcα2-6Gal linkages and significantly increases recovery of recent H3N2 strains from patient samples (64). Although glycomic analyses of MDCK cell lines are not yet available, it is clear that simply increasing the amount of NeuAcα2-6Gal receptors on these laboratory hosts appears sufficient to achieve enhanced receptor binding and infection by human influenza viruses.

### Compensatory changes in N2 NA activity that promote receptor binding

It is widely accepted that IAVs require a balance between the receptor-binding avidity of HA and the receptor-destroying activity of NA for optimal binding and entry into host cells, avoidance of becoming trapped by mucins, and for release of newly synthesized virus particles from the infected cell (10, 11, 12, 13, 14). In general, this means that low-avidity HAs need to be coupled with low-activity NAs, and vice versa (10, 11, 13, 14). Due to the very low avidity of recent H3 HAs for shorter α2-6 glycan receptors, propagation in MDCK cells frequently results in a low-activity N2 NA mutation, D151G, located within the catalytic site (65, 66, 67, 68). D151G dramatically reduces NA activity, leading to NA replacing some of the role of HA for binding to glycan receptors on MDCK cells and mediating virus hemagglutination of erythrocytes in HAU titering assays (65, 68). On glycan arrays D151G NAs bind both α2-3 and α2-6-linked sialosides, but show strong preference for α2-3 linkages, in keeping with their preference for these glycans as substrates (66). Importantly, when H3N2 viruses with this mutant NA are propagated on cultured human airway epithelial (HAE) cells, they have been shown to quickly revert to wild-type (D151) (69), providing direct evidence that this variant is artificially selected for through passage in MDCK cell laboratory hosts. That D151G reverts to wild-type with restored ability to hydrolyze HAE glycan receptors suggests that the driving force underlying its selection comes from a requirement to balance the avidity of HA for receptors on HAE cells, the natural host (69).

Although the NA D151G mutation is clearly laboratory-induced and does not occur in natural isolates, a natural mutation resulting in a comparable hemagglutination phenotype in circulating H3N2 NAs has been identified in the same region (50). Between 2005 and 2008, human IAV N2 NAs underwent a gradual selection, steadily incorporating an H150R variant that has now become fully dominant in subsequent strains through to the present day. Like laboratory-induced D151G variants, some naturally occurring H150R H3N2 viruses have been shown to hemagglutinate both turkey and human erythrocytes through NA-mediated binding, which can be distinguished from HA-mediated hemagglutination through addition of the NA inhibitor oseltamivir (50). While this clearly impacts hemagglutination assays in the laboratory, particularly with regard to distinguishing hemagglutination by the HA or NA, an important question that remains to be determined is if this sialic-acid-binding function of the H150R N2 NA is biologically relevant to infection and/or human-to-human transmission of recent H3N2 strains.

## Receptor specificity of avian influenza viruses that cause sporadic zoonotic infection of humans

Over the last 20 years, several avian influenza viruses including H5N1, H7N9, H6N1, and H10N8 have been documented to cause sporadic zoonotic infections among humans, raising the potential for host adaptation and human-to-human transmission as a novel pandemic outbreak. While the HAs of such viruses isolated from infected human cases typically retain strong specificity for avian-type receptors, some have demonstrated specific signs of potential adaptation to human receptors. One significant example of this is found in H7N9, where, in recent (particularly fourth and fifth) waves, human isolates of avian viral infections have been found to be highly dominant for the Q226L mutation associated with “humanized” receptor specificity in both H2N2 and H3N2 pandemics (70). However, various studies working both *in vitro* and *in vivo* have now demonstrated that the potential for sustained human-to-human transmission, measured by proxy either through airborne infections in the ferret model or through conversion to human receptor specificity on glycan microarrays, is likely both complex and individual to different subtypes, requiring distinct combinations of multiple different adaptive mutations. In this section, we review current knowledge on likely adaptations to support human transmission in prominent avian virus subtypes, as well as evidence for potential selection in nature.

### H5N1

Current H5N1 strains emerged in avian species in China in the mid-1990s and are both highly contagious and extremely pathogenic in birds. Initial human H5N1 cases were detected in 1997 (71, 72), and WHO now reports over 860 documented human H5N1 infections between 2003 and 2019, with a mortality rate of approximately 50% (https://www.who.int/influenza/human_animal_interface/H5N1_cumulative_table_archives/en/, June 2020), leading to heightened concern about a potential pandemic (by comparison, 1918 pandemic H1N1 estimated case mortality is only approximately 10% (2)). Fortunately, however, most human H5N1 infections to date have been associated with acute exposure to live poultry, and zoonotic strains have shown very limited adaptation for human receptor binding or human-to-human transmission, despite persistent low-frequency introductions for more than two decades. Potential for an H5 pandemic has thus prompted great research into circulating viral mutants that might confer human adaptation, particularly within HA. To date, natural variants with potential to alter receptor specificity have been reported at more than 20 individual H5 amino acid positions, with many more combinations of both natural and engineered mutants also studied (reviewed in detail in ref. (73)). Thus far, however, no naturally occurring H5N1 viruses (whether human or avian isolates) have demonstrated the strong α2-6 receptor specificity associated with pandemic outbreaks or sustained human-to-human transmission (73). While natural H5 variants at numerous positions, including 133, 137, 138, 144, 155, 186, 187, 190, 192, 193, 196, and 197 (all H3 numbering), do appear to increase binding to human-type receptors, all examined isolates have been shown to retain equal or dominant binding to α2-3-linked glycans, suggestive of only partial adaptation (74, 75, 76, 77, 78). *In vitro* protein engineering studies using recombinant H5s (79, 80), together with *in vivo* experiments aimed at directly evolving H5N1 viruses (77, 81, 82), have shown that the pathway toward full “humanization,” resulting in either fully altered receptor specificity or full airborne transmission in ferrets, is in fact quite complex and requires multiple adaptive changes (73). In all cases, complete switches to human receptor specificity and/or full airborne transmission could only be achieved in HA backgrounds harboring preadaptive mutations, including Q226L plus either engineered N224K (81) or G228S (77, 79, 80, 82) (none of which circulate at high levels in native H5 viruses), complemented either by further naturally occurring variants in the case of recombinant HAs (80) or by serial passage in ferret hosts in the case of evolved virus models (77, 81, 82). Three elegant directed evolution studies published in 2012 by Imai *et al.* (81), Herfst *et al.* (82), and Chen *et al.* (77) all further demonstrated that in addition to HA RBS mutations, H5N1 viruses with pandemic potential likely required a combination of: HA stem-domain variants to increase stability (T318I (81) or H107Y (82)), removal of a head-domain glycosylation site (all studies (77, 81, 82)), and either specific humanizing mutations within certain gene segments (E627K variant required within the native PB2 (82)) or complete replacement of entire avian genes with at least one human IAV gene outside of HA (recovery with Cal/04 (pdmH1N1) internal genes (81) or recovery with a substituted human N2 gene (77)). Together, all of these observations are suggestive of a large barrier to entry and sustained infection of H5(N1) in humans without significant adaptive mutation(s) or recombination. Furthermore, WHO data shows there has been a substantial reduction of H5N1 human infections since 2015 to 2016, thus strongly reducing the potential for such evolution to occur and likely lowering the risk of a new pandemic.

### H7N9

Human infections with avian H7N9 viruses were first reported in 2013 (70) and, similar to H5N1, have been strongly associated with acute exposure to infected live poultry. Despite only recent appearance, human H7N9 infections have spread rapidly, with over 1500 confirmed cases and over 600 deaths worldwide between 2013 and 2019 (https://www.who.int/docs/default-source/wpro---documents/emergency/surveillance/avian-influenza/ai-20190830.pdf?sfvrsn=30d65594_34, https://www.who.int/csr/don/05-september-2018-ah7n9-china/en/, June 2020). Also similarly to H5N1, zoonotic avian H7N9 isolates from human patients have typically retained strong binding to α2-3-linked receptors, suggesting only poor or partial adaptation to human hosts; however, given the rapid spread of human cases, and prior reports that H7 genes in alternate subtypes, including H7N7 and H7N2 (83, 84), can bind strongly to human receptors, close attention has been paid to circulating H7N9 strains due to the possibility of heightened pandemic potential. H7N9 strains have entered the human population through a series of seasonal epidemic outbreaks or “waves,” with the most severe fifth wave in 2017 featuring the largest number of cases, widest geographic spread, and emergence of HPAI (highly pathogenic avian influenza) strains. Emphasizing both the significance of the outbreak and growing global resources and research maturity in the influenza field, analyses of early first-wave H7N9 strains were particularly rapid, with numerous high-quality studies published within months of the first human infections. Early viral isolates were shown to contain numerous high-risk variants, including PB2 E627K (enhanced polymerase activity/virulence in mammals (85)), NA R294K (oseltamivir resistance (86)), and HA G186V and Q226L (receptor specificity switch mutations associated with pandemic viruses) (70, 87, 88, 89, 90). Despite containing HA adaptive variants, solid-phase binding assays, including glycan microarrays, demonstrated early human H7N9 strains to have mixed receptor specificity and, in most cases, retained strong predominant binding to avian α2-3 receptors (87, 88, 89, 91, 92, 93, 94, 95, 96). Several crystal structures in complex with both avian and human receptor fragments revealed receptor-binding modes most in common with avian-adapted HAs, with human receptor cocomplexes showing a noncanonical conformation for the receptor glycan when compared with human HA counterparts (87, 89, 95, 96). Pathogenesis studies revealed that while new H7N9 viruses did appear well adapted for infection and replication in various mammalian hosts, including mice (88, 94), ferrets (88, 91, 94, 97, 98), pigs (97), and nonhuman primates (94), most isolates could not be efficiently transmitted *via* airborne respiratory droplets in the ferret model (88, 91, 94, 97, 98) (typically only one out of three recipients) and were therefore unlikely to lead to significant human-to-human transmission. Since first-wave 2013 strains, H7N9 viruses have continued to circulate, becoming enzootic in birds and causing sporadic human infections in seasonal epidemic waves with little significant change in viral properties (99) until a fifth epidemic in 2017 that led to the emergence of HPAI H7N9 (100). Nonetheless, despite both its wide geographic spread and record numbers of human infections, fifth-wave human H7N9 viruses, including highly pathogenic strains, have shown little further adaptation to human receptor binding or airborne transmission in ferrets (100). Similar to earlier studies for H5N1, the combination of a potential (but poorly defined) pandemic threat together with evident partial human adaptation has led to increased interest in further adaptive mutations that might be required for H7N9 viruses to fully cross the species barrier into humans. Studies of whole-virus models revealed variants at positions 104, 219, 226, 228, and 387 (H3 numbering) to have positive adaptive effects on HA stability (both thermal and pH), fusion pH, and receptor binding (101). Interestingly however, and similar to H5, introduction of G228S into the native L226 background, while increasing binding to α2-6 receptors (101, 102), was not sufficient to result in a switch in binding preference or to reduce binding to avian receptors (101). Glycan array analysis of recombinant H7 proteins and engineered variant combinations have subsequently revealed, again similarly to H5, a large barrier to full human receptor-binding adaptation, with that at least two further variants, in addition to G228S, being required for complete α2-6 receptor specificity (103). Early studies on adaptation of human-isolated H7N9 viruses in ferrets suggest that while these strains are comparatively free to evolve within avian species, replication within mammals imposes a very narrow genetic bottleneck, often preventing or strongly inhibiting selection of favorable adaptive mutants (104).

### H6N1, H9N2, and H10N8

While H5N1 and H7N9 represent the vast bulk of zoonotic avian IAV infections in humans, they are far from the only avian subtypes that have shown potential to cross the species barrier, with confirmed infections or evidence of exposure now recorded for at least a half-dozen further viruses, including but not limited to: H5N2 (105), H5N6 (106), H6N1 (107), H7N1 (105), H7N3 (108), H7N7 (109), H9N2 (110), and H10N8 (111). In this section, we briefly review a selection of these for which detailed receptor specificity and structural analyses have been carried out.

H6N1 IAV strains circulate primarily in avian species in East Asia, Europe, and North America. To date, just a single confirmed case of H6N1 in humans has been reported (107), with seroprevalence studies of poultry workers suggesting similarly low rates of exposure (112), indicative of likely subclinical disease in most cases and thus poor adaptation to human infection. Subsequent receptor-binding and structural analyses comparing the human H6N1 infection strain with circulating avian H6 counterparts from the same period revealed two noncanonical receptor-binding variants: V190 (previously unknown) and S228 (classical H2/H3 humanizing) (113, 114, 115). As expected, avian H6 HAs showed strong α2-3 avian-type receptor specificity, while human isolates featured some increased α2-6 binding, though at best overall mixed specificity (113, 114, 115). Interestingly, and in contrast to H5N1 & H7N9, an *in vitro* receptor-binding evolution study by de Vries *et al.* (115) demonstrated that complete human-type receptor specificity switching in the human H6 isolate could be achieved by introduction of just a single variant, G225D (associated with H1 pandemic viruses), albeit at an apparent cost to avidity. However, given that G225D is not found to naturally occur in more than 600 H6N1 isolates currently present within the GISAID database (Global Initiative on Sharing All Influenza Data; www.gisaid.org), and overall apparent low infection potential in humans, H6N1 viruses likely do not pose a major pandemic threat.

H9N2 viruses circulate in avian species, particularly poultry, mainly within Russia, China, Southeast Asia, the Middle East, and North Africa. Similar to H6N1, human infection with H9N2 most frequently leads to mild disease; however, transmission appears to occur substantially more frequently (approximately 70 confirmed cases recorded since 1999 [https://www.who.int/influenza/human_animal_interface/HAI_Risk_Assessment/en/, June 2020] [116]), with the majority of cases also appearing in younger people (116). H9N2 is a large and complex IAV subtype divided into three clades: G1 (Eastern & Western lineages), BJ94, and Y439 (117), with human infections arising from strains present in all three clades, though mostly G1 and BJ94 (116). Early H9N2 viruses largely corresponded to classical avian-type receptor-binding genotypes including E190 and Q226 (H1 and H2/H3 key determinant positions, respectively); however, subsequent and contemporary strains now feature substantial variability, including widespread circulation of humanizing Q226L mutants, particularly within G1 and BJ94 clades (118, 119). While early studies have suggested that Q226L-bearing H9N2 viruses lead directly to significant binding of human-type receptors (118, 120), similar to H5 viruses, receptor specificity within H9 is complex. A range of amino acid variants at positions, 155, 183, 190, 193, 226, 227, and 228, have all been shown to contribute to binding to a variety of glycan epitopes including α2-3, α2-6, Sialyl-Lewis-X, and sulfated sialoglycans, influencing properties such as tissue tropism, host range, and transmission (119, 121, 122, 123, 124). We have recently shown that while naturally occurring H9N2 variants at position 226 generally correspond to Q, L, or M, substantial plasticity leading to viable receptor binding remains possible, with many variants retaining substantial specificity for human-type receptors (125). Given that H9N2 strains have already been shown to be capable of substantial reassortment with other circulating avian IAV subtypes, notably H5N1 (126), H7N3 (127), and H7N9 (128), careful future surveillance for potential pandemic risk is warranted.

By comparison, H10N8 strains are relatively rare, circulating predominantly in wild aquatic birds, though also somewhat in poultry, and largely isolated to mainland China (129, 130). Similar to H6N1, H10N8 infections in humans are extremely rare, with only three confirmed cases to date; however, two of these resulted in fatal disease (111, 131). Both the human H10N8 isolate and contemporary avian strains conserve canonical avian-type amino acid variants: E190, G225, Q226, and G228, and have been shown to maintain strong specificity for avian-type receptors, with introduction of classical H1 and H2/H3 specificity switching mutations at these positions resulting in no change or complete loss of receptor-binding function (132, 133). Together with H7 and H15, H10 HAs form a small phylogenetic cluster within IAV HA genes containing a unique two-amino-acid insertion in the 150-loop, between positions 158 and 159 (standard H3 numbering), which is absent in all other human and avian HAs (133). Presence of this additional steric bulk at a position immediately above the H10 RBS appears to negatively impact potential human-type receptor binding, where the underlying glycan chain lies in close proximity to this position (see further), with *in vitro* receptor-binding evolution studies showing removal of at least one of these additional side chains to be required, in addition to introduction of Q226L-G228S, to achieve full human-type receptor specificity switching (133). Requirement for such substantial changes both within and around the H10 RBS, coupled with so few human infections to date, likely indicates a significant barrier to human adaptation or even widespread zoonotic transfer.

## The structural basis for avian- and human-type receptor specificities

**Figure 6:**
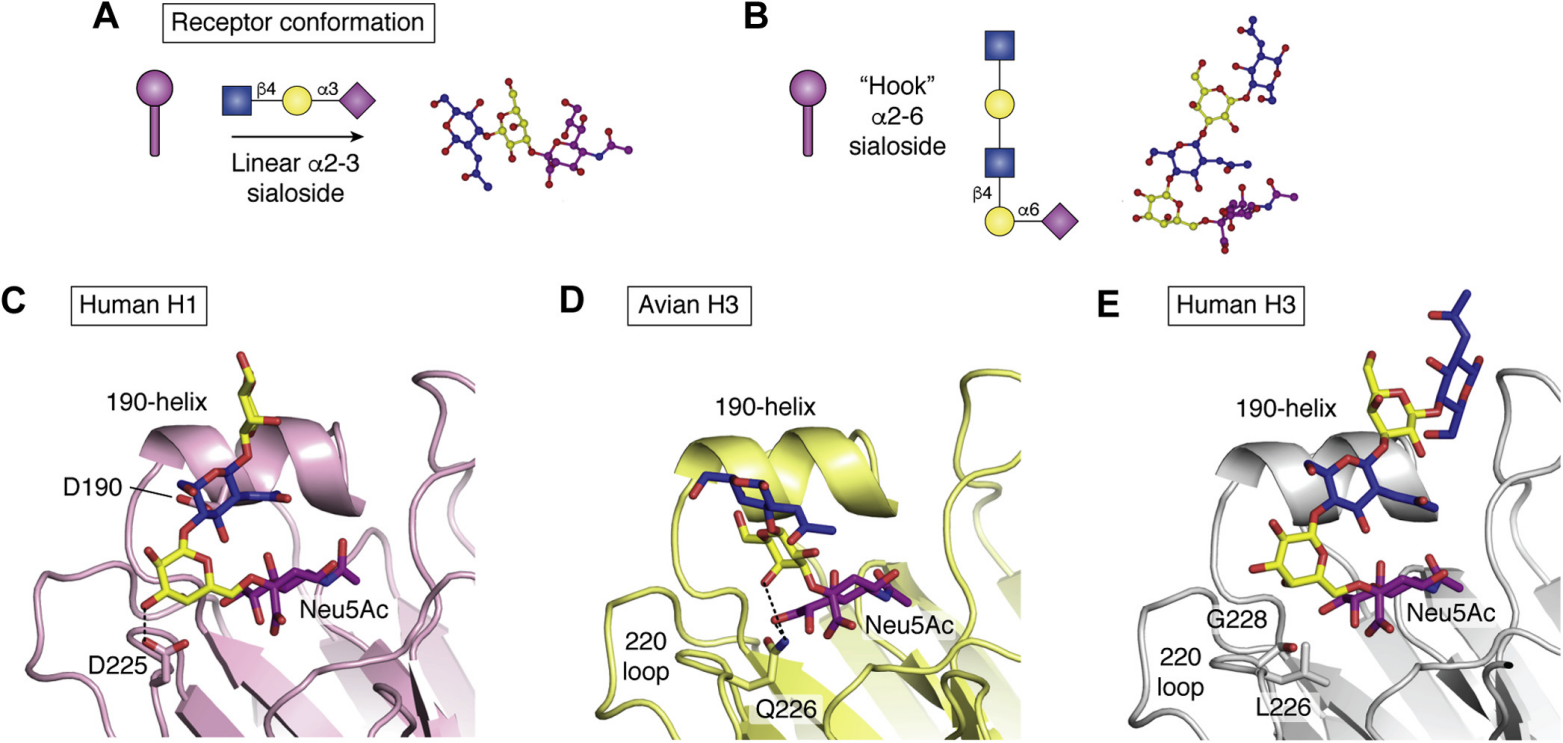
**Receptor-binding adaptations are dictated by the conformation of receptor itself.***A*, cartoon and isolated receptor structures showing respective low-energy linear and “hook”-shaped conformations of α2-3 and α2-6 sialosides, respectively. *C*–*D*, human-adaptive variants favor accommodation of the α2-6-linked receptor conformation and lead to hindrance of linear α2-3 receptors. *C*, the smaller D190 side chain increases stability and reduces clashing with human-type receptors, while D225 adds a new H-bonding interaction. *D*, Q226 favors binding of galactose in the linear avian-type conformation through H-bonding interactions. *E*, mutation of Q226 to leucine (L) removes favorable α2-3-binding H-bonds while the new hydrophobic side chain hinders sugars lying directly over this position. Panel (*A*) assembled using CCP4Mg (148), panels (*C*–*D*) assembled in Pymol (Schrodinger LLC) using PDB IDs: 1RVZ, 1MQM, & 6TZB.

While transition to sustained human infection and transmission is complex and multifaceted, requiring adaptation of multiple genes and viral properties, the human–avian influenza species barrier for receptor binding of HA can be defined simply by the varying prevalence of alternate terminal sialic linkages on glycan receptors in the upper respiratory tract of these species (3, 4). Within this context, avian species contain predominantly α2-3-linked sialic acids, presumably through upregulated activity of α2-3-sialyltransferases, while the human upper airway tends toward α2-6 for likely similar reasons (74). However, while the activities of these enzyme families may appear similar, with overlapping competition for the same/similar β-galactoside substrates, their respective products confer strongly differing properties on the final glycan structure. Early structural NMR studies (134, 135, 136) have shown that even in free solution, α2-3 sialosides are comparatively rigid molecules, imposing an approximately linear conformation along the length of the glycan chain (Fig. 6*A*). Conversely, bond formation through a primary alcohol to create the α2-6Gal linkage in human-type receptors results in increased flexibility, leading to a low-energy solution conformation where the α2-6-linked sialoside appears folded back toward galactose, stabilizing its position and creating an overall “hook” or “closed umbrella” conformation (134) (Fig. 6*B*). Such structural variances are thus important for HA receptor binding, since estimated *K*_d_ values for an individual HA RBS interacting with a single sialylated receptor are exceptionally weak (137, 138), meaning that influenza viruses rely on highly multivalent engagement of multiple HA trimers with multiple glycan receptors on the surface of a cell to successfully initiate infection (139). Binding of HAs *via* a large multiplicity of individually weak interactions therefore typically leads to minimal distortion of the receptor within the RBS, a feature commonly observed in numerous HA crystal structure complexes, where bound receptor glycans frequently appear in their respective low-energy linear or hook-shaped conformations (140, 141). These alternate and comparatively static structures of the avian and human glycan receptors thus define the evolutionary target for human adaptation, wherein variants at specific locations within the RBS that are able to reduce recognition of linear α2-3 sialosides, and/or enhance recognition of hook-shaped α2-6 glycans, play key roles in receptor specificity switching.

### Structural variants underlying avian HA adaptation to human-type receptor specificity

HA variants that contribute toward preferential binding of human α2-6 receptors arise in two general locations within the RBS, according to the structural differences/requirements between the two glycan conformations. Variants within the 220-loop, which sits immediately beneath the glycosidic linkage between sialic acid and galactose in both human and avian HA-receptor structures (sialic acid is coordinated in a conserved position regardless of receptor specificity), influence whether linkages belonging to either linear (α2-3 avian-type) or hook-shaped (α2-6 human-type) conformations are preferentially accommodated. For example, compared with precursor avian-like H1 structures, the evolution of G225D at the base of the RBS in human pandemic H1N1 viruses adds a novel, bulky side chain providing steric hindrance close to underlying sugar residues beyond the NeuAcα2-3Gal glycosidic bond and creating an additional H-bond that preferentially engages galactose when presented in the “folded-back” conformation of human α2-6 receptors (28, 30) (Fig. 6*C*). Beyond the 220-loop, humanizing variants typically fall within structures above the RBS, including within the 190-helix. These regions gain increased importance in human-adapted HAs since the underlying sugar molecules within the α2-6-linked human receptor glycan chain lie in close proximity to the upper surface of HA (Fig. 6, *C* and *E*), thus variants here act to increase stability and remove larger side chains that might potentially lead to steric interference of human receptor engagement. This is exemplified by the evolution of E190D in human pandemic H1N1 viruses, which maintains a negatively charged amino acid but leads to shortening of the overall side-chain length by one carbon. Within pandemic human H1 structures, D190 appears more recessed within the RBS, likely stabilizing the overall position of the 190-helix and allowing for more optimal accommodation of the α2-6-linked glycan chain over the top of this structure (28, 30) (Fig. 6*C*).

Such a pattern of evolution appears to hold well in human H2N2 and H3N2 pandemic strains, where Q226L and G228S variants (also implicated in humanization of H5 and H7) influence specificity by adding hydrophobic and/or bulky amino acid side chains below the glycosidic bond, reducing the binding preference for linear α2-3 receptors, which lie directly over these positions, and favoring the accommodation of α2-6 linkages where the glycan chain folds back toward the top of HA (Fig. 6, *D*–*E*). Examples of receptor specificity adaptations beyond the RBS have been reported through *in vitro* evolution experiments where receptor specificity of candidate H7 HAs derived from zoonotic human infections could only be switched to full human-type receptor binding through specific triple mutant combinations incorporating variants at positions 186, 193, 224, and 228 (103). Within H10 and H15, natural insertions of additional bulky amino acid side chains into the 150-loop prevented full conversion to human specificity, even in the presence of variants at 193, 226, and 228, and were required to be removed or substantially mutated to achieve exclusive α2-6 sialoside binding (133, 142).

Further to roles purely in receptor engagement, variants that contribute to specificity switching often lead to changes in additional HA properties, such as immune recognition, particularly within human influenza viruses. While residues in the 220-loop do not fall within recognized antigenic sites, and are not typically thought to be immunogenic (143), amino acids within the 190-helix and 150-loop form a major part of antigenic site B (in H3) and are strongly associated with antibody recognition (144, 145). Adaptive changes required to accommodate glycan chains with terminal α2-6 linkages outside of the RBS, and that would likely impact antigenicity within these more distal regions, have already been described and include: K193T (103, 146) (H5 and H7) and K158aA or ΔK158 (133, 142) (H10 and H15), as well as N158D/K or T160A (H5), leading to the removal of a glycosylation site at position N158 (147). Thus, adaptation to human-type receptor specificity results in an expansion of the glycan-interacting surface of HA and strongly links receptor-binding properties to those of antigenicity around the top of the HA head domain.

Finally, engagement of glycan receptors from above the top face of the HA molecule, as required for α2-6-linked receptor binding, opens the possibility of more complex receptor-binding interactions. Recently, we have demonstrated that contemporary H3 viruses have evolved to select for elongated α2-6-sialoside receptors with at least three N-acetyllactosamine repeats beneath the terminal sialoside (49) (Fig. 5). That early and late H3s show similar receptor-binding modes within the core RBS suggests that variants at more distal positions are likely responsible for this new phenotype. Notably, elongated receptors are required to loop substantially over the upper surface of HA, likely leading to evolution of increased protein–glycan contacts and thus increasing the likelihood that such variants will also contribute to antigenicity. While extended glycan receptors appear rare, molecular dynamics simulations have revealed a possible rationale for this evolution of receptor specificity through the potential enhancement of binding avidity, where a single extended biantennary N-glycan receptor could engage in a bidentate interaction with two HA protomers within the same trimer simultaneously (49). Circumstantial evidence for enhanced receptor binding has been revealed through observations that recent H3N2 strains have been able to undergo major antigenic changes through evolution of a novel glycosylation site at position N158 (57), an alteration previously shown to be incompatible with human receptor specificity in H5N1 viruses (147). Due to their extremely narrow receptor specificity, contemporary H3N2 viruses have now become extremely difficult to grow within standard laboratory cell line hosts and undergo significant adaptative changes when passaged in eggs, strongly impacting effectiveness of the H3N2 component of seasonal vaccines (57). The continued antigenic and receptor-binding evolution of H3N2 viruses, potentially leading to increasingly severe seasonal outbreaks, now represents perhaps the most imminent threat of influenza to human health, and further research is required to fully understand and develop long-term solutions to this growing problem.

## Summary and conclusions

During the last century, since the outbreak of the 1918 influenza pandemic, knowledge and understanding of basic influenza biology, and particularly how potential pandemic strains pose risks to health through adaptation and/or reassortment, have grown at an amazing pace. While it is generally believed that acquisition of human receptor specificity is one, among several, prerequisites for efficient infection and transmission among humans, and for respiratory droplet transmission within animal (particularly ferret) models, the true strength of receptor specificity as part of the host species barrier remains poorly defined. To date, only very few studies have been able to examine effects of specifically altered properties within reverse genetics models of avian influenza viruses that pose a threat to human health, and even these have been largely confined to H5N1. Fortunately, both these and other *in vitro* studies focusing on recombinant avian HAs have suggested that the barrier to full adaptation and/or acquisition of full human receptor specificity is high, likely requiring the coevolution of several specific humanizing variants within a single genetic background. Analysis of the three influenza pandemics occurring within the last century, subsequent to 1918, suggests that acquisition of several such properties within a short period of time is more likely to occur *via* reassortment of numerous partially adapted strains within hosts such as poultry or swine that harbor large, diverse ranges of viral subtypes. Thus, prediction or preparation for a potential pandemic is made more difficult since the properties of emerging novel strains may only partly correspond to that of avian/swine-adapted progenitors. Nonetheless, with a growing population of avian influenza strains that appear at least “pre-adapted” for binding to human-type receptors (circulation of Q226L variants is increasingly common), and with various other questions still open, further studies to directly correlate receptor binding with infection and transmission in cell culture, mice, and ferret models remain important. For example, do observations that numerous additional mutations are required for receptor specificity switching in H5N1 also apply to other avian subtypes such as H7N9? Given recent observations that NA-dependent binding is now possible in human H3N2, and prior knowledge of HA/NA balance, what is the contribution of NA to potential host adaptation? Finally, although human infections with avian influenza strains have slowed in recent years, how do we continue to monitor or assess potential future evolution of receptor specificity both for avian and human strains (*e.g.*, H3N2)? Regardless of outcome, providing answers as to the contribution of receptor specificity to cross-species viral adaptability represents a significant goal in global influenza surveillance efforts and our understanding of pandemic potential.

## Conflict of interest

The authors declare that they have no conflicts of interest with the contents of this article.
